# Side-Scan Sonar Image Segmentation Based on Multi-Channel CNN for AUV Navigation

**DOI:** 10.3389/fnbot.2022.928206

**Published:** 2022-07-19

**Authors:** Dianyu Yang, Chensheng Cheng, Can Wang, Guang Pan, Feihu Zhang

**Affiliations:** School of Marine Science and Technology, Northwestern Polytechnical University, Xi'an, China

**Keywords:** side-scan sonar, segmentation, CNN, large kernel, multi-channel

## Abstract

The AUV (Autonomous Underwater Vehicle) navigation process relies on the interaction of a variety of sensors. The side-scan sonar can collect underwater images and obtain semantic underwater environment information after processing, which will help improve the ability of AUV autonomous navigation. However, there is no practical method to utilize the semantic information of side scan sonar image. A new convolutional neural network model is proposed to solve this problem in this paper. The model is a standard codec structure, which extracts multi-channel features from the input image and then fuses them to reduce parameters and strengthen the weight of feature channels. Then, a larger convolution kernel is used to extract the features of large-scale sonar images more effectively. Finally, a parallel compensation link with a small-scale convolution kernel is added and spliced with features extracted from a large convolution kernel in the decoding part to obtain features of different scales. We use this model to conduct experiments on self-collected sonar data sets, which were uploaded on github. The experimental results show that ACC and MIoU reach 0.87 and 0.71, better than other classical small-order semantic segmentation networks. Furthermore, the 347.52 g FOLP and the number of parameters around 13 m also ensure the computing speed and portability of the network. The result can extract the semantic information of the side-scan sonar image and assist with AUV autonomous navigation and mapping.

## 1. Introduction

As interest in the underwater has increased in recent years, the autonomous navigation capabilities of AUVs, the primary vehicle for underwater exploration, have received widespread attention from the scientific and industrial communities. Allotta et al. (2016) present an innovative navigation strategy designed explicitly for AUVs based on the Unscented Kalman Filter (UKF) because of the problem that GPS cannot be used underwater. Similarly, Shao et al. (2016) Adaptive Extended Kalman Filter (AEKF) algorithm to AUV is applied to solve the problem that the Kalman Filter noise covariance matrix is complicated to determine. They put forward their solutions to the problem of error accumulation in inertial navigation. However, their research ideas are relatively limited, and they do not consider using other sensors for assistance. Song et al. (2020) a neural network-based AUV navigation method for fast-changing Environments, employs the neural network to predict pitch angles accurately to compensate for accumulated errors in INS. The author uses the neural network Angle prediction method to compensate for the accumulated error well. However, the prediction result is still far from the ideal value because the neural network design is relatively simple. Franchi et al. (2020) means to develop an underwater navigation system that does not rely on a DVL. Instead, an algorithm is developed to obtain linear velocity based on forward-looking sonar. Song et al. (2016) use side-scan sonar images for image registration to improve the accuracy of AUV navigation. They all use sonar sensors to obtain additional environmental information to compensate for navigation accuracy, which is better than relying solely on inertial navigation. However, the processing method of sonar data is rough, and all the image information is not thoroughly used. Yang et al. (2019) designed a neural network to extract features of side-scan sonar images and perform terrain matching to assist AUV navigation. Their model has dramatically improved accuracy compared with previous work and can effectively extract image features for similarity judgment. However, their limitation lies in using ROI scanning to extract local features without establishing a more extensive range of global feature matching. Siantidis (2016) used the particular target detected in the side-scan sonar image to assist SLAM loopback detection and improve the autonomous navigation capability of AUV. However, he uses the method to manually design the feature extraction algorithm to search the target, which leads to low detection accuracy. Petrich et al. (2018) presented the development and experimental validation of a side-scan sonar-based self-localization algorithm for AUVs. It was shown that multimodal feature extraction yields diverse feature maps that lend themselves to robust feature matching. However, they only used the acoustic echo characteristics of the side-scan sonar, not the image information.

After the development of AUV navigation technology, the current mainstream direction has changed from the simple dependence on inertial navigation and filtering algorithms to the combination of multi-sensor information and intelligent algorithm. Therefore, as an idiomatic sensor with high underwater accuracy, side-scan sonar has also received more attention (Reed et al., 2003; Acosta and Villar, 2015). As an imaging sonar, the side-scan sonar can receive acoustic signals reflected from underwater targets and, after analysis, image objects on sonar images according to the distance between reflected objects and sonar (Zhang et al., 2019). The sonar image obtained in this way has the characteristics of low resolution, fuzzy target area, complex edge information, and noise interference, which brings difficulties to the accurate segmentation of the target area of the sonar image (Wang et al., 2022). So sonar image recognition largely depends on professionals' specially designed filtering algorithms and artificial judgment. However, the underwater environment is complex, and there are many interference factors, so it is challenging to design a filtering algorithm that can play a good role. In addition, artificial judgment also consumes a lot of time and energy. It is necessary to design an algorithm model that can extract features and identify them by itself.

In recent years, image classification and segmentation models based on deep learning have made great progress, among which VGG-Net (Simonyan and Zisserman, 2014), GoogLeNet (Szegedy et al., 2014), and Resnet (He et al., 2015) based on the convolutional neural network have achieved good results in classification tasks of many camera image data sets. Image segmentation models based on FCN (Long et al., 2015), U-Net (Ronneberger et al., 2015), PSPNet (Zhao et al., 2017), and other models have also attracted the attention of many researchers. The detection and segmentation model based on deep learning can extract features and classify results by itself, which helps to alleviate the difficulty of feature extraction algorithm design and reduce the level of human participation in the recognition process. Therefore, there has been more and more research on underwater sonar image recognition and segmentation using deep learning models in recent years.

Song et al. (2017) proposed a side-scan sonar image target detection method based on the FCN model (FCNs). They use deep CNN, which is derived from FCN, to segment images of SSS into three parts: highlight areas with objects, regions of shadow, and sea-bottom reverberation areas, then use MRF to post-process the results. However, there is no innovation in the model, and the validation index of the experimental part is relatively single, which makes the results of the reference significance is low. Chen and Summers (2017) proposed an unsupervised CNNs model for synthetic aperture sonar image segmentation. The method demonstrates a semi-supervised method for utilizing unlabeled or weakly labeled imagery for joint training with few densely labeled images for semantic segmentation. Wu et al. (2019) proposed a practical convolutional neural network structure (ECNet) for target region segmentation of side-scan sonar images. The ECNet consists of an encoder network to capture context, a corresponding decoder network to restore full input-size resolution feature maps from low-resolution ones for pixel-wise classification, and a single stream deep neural network with multiple side-outputs to optimize edge segmentation. Its disadvantage is that the sonar image data size is small. Moreover, it only targets the task of binary classification. It does not involve large-scale images and multi-classification closer to the actual situation. Huo et al. (2020) proposed a semisynthetic data generation method for producing sonar images of airplanes and drowning victims, which uses optical images as input, and combines image segmentation with intensity distribution simulation of different regions, then use the CNN model to complete the classification task. However, the network is more inclined to identify specific targets rather than the whole segment. At the same time, it has strict requirements on the clarity and Angle of the target. The segmentation result is established in a data set that has been accurately marked, which reduces the robustness of the model. Zhou et al. (2020) proposed a sum-modified Laplacian energy filtering with a CNN model for image fusion of side-scan sonar. This paper proposes a sum-modified Laplacian energy filtering and improved dual-channel pulse coupled neural network for image fusion of side-scan sonar in non-subsampled contourlet transform. However, the author also points out that the proposed frequency-based segmentation criterion is not perfectly applicable to all sonar images, proving the limitations of the artificial feature extraction algorithm for global feature extraction. Ding et al. (2017) used SAILFISH AUV to collect 180*36 Pixels side-scan sonar images and classify them using ERF. Li et al. (2019) designed a real-time sonar image segmentation algorithm based on ENet and MRF. Petillot et al. (2002)implemented real-time pipeline detection using AUV with side-scan sonar and multi-beam echo-sounder. McConnell et al. (2022) used an AUV equipped with side-scan sonar to construct an underwater obstacle grid similar to an aerial map by using the CNN network and realized AUV underwater SLAM based on it. Yin et al. (2021) Studied the underwater SLAM mission based on AUV, Monocular camera, STEREO Camera, Single-beam sonar, multi-beam sonar, side-scan sonar, and Laser. The conclusion is that acoustic devices still hold a great advantage, but optical sensors can be considered a supplement to accuracy. Połap et al. (2022) proposed an algorithm based on a neural network to achieve Object signaling, low-quality areas detection, and seabed line extraction tasks. This network is also aimed at sonar images' small target recognition task and does not involve the large-scale downstream task. Zhu et al. (2017) proposed a model to extract target features by a convolutional neural network (CNN) operating on sonar images and then classified by a support vector machine (SMV) that is trained based on manually labeled data. The network model is relatively simple and based on the matched filter designed manually to improve the network performance, which also leads to the insufficient feature extraction ability of the network.

It can be found that current relevant studies mainly focus on the target detection of small-scale sonar images, and there are not too many studies focusing on large-scale background segmentation tasks. At the same time, it is seldom involved in multi-classification tasks, which are primarily binary classification tasks with the help of manually designed feature extraction algorithms. This paper proposes a CNN model that aims at side-scan sonar image segmentation to address these questions. Due to the difficulty obtaining sonar image data, the designed neural network model needs to control the depth and number of parameters to avoid over-fitting. Meanwhile, as the color richness of the sonar image is not as good as that of the camera image, it is relatively simple. The information contained in different color channels should be considerably different. Feature extraction focusing on a single channel should be helpful to improve classification accuracy.

The main contributions of this paper are as follows:

1. We used the AUV developed and manufactured to collect the actual side-scan sonar data in the lake area, established a data set, manually annotated the data, and conducted network training for multi-classification background segmentation.
2. We separate the RGB channels of the input image for feature extraction and then concatenate them after the multi-layer network. Then we increased the convolution kernel size to 7 × 7, which proved effective in sonar images with a larger size. Finally, we add a parallel feature extraction channel using a small convolution kernel and concatenate the output features of different levels with the output of the decoder to compensate for the insufficient feature extraction problem of large convolution checking small targets.
3. We use our test set to test the network model's performance and compare our model's performance with that of several other classic segmentation networks. At the same time, we experiment with the influence of different model modules on the performance, and the conclusion is that our model has a good segmentation effect.

The rest of this paper is organized as follows. In Section 2, the related works are introduced. In Section 3, our model is described in detail. The experiments on the sonar image dataset are implemented in Section 4, and the conclusion is described in Section 5.

## 2. Related Work

In this section, the source of design ideas, which are principles of CNNs, the structure of U-net, and the effect of the size of kernel size, are introduced.

### 2.1. Principles of CNNs

Neural network models with CNN have been completed by Lecun and Bottou (1998) and carried forward by AlexNet (Technicolor et al., 2012). The classical CNN structure generally comprises the convolution layer, the pooling layer, and the activation function layer. The data only flow in two directions: forward and backward. The image first passes the convolution layer inside the network after entering the network model in the forward direction. After each convolution layer, the image becomes a feature graph of higher dimensions. After the pooling layer, the image size becomes smaller, equivalent to a parameter reduction and further feature extraction. Finally, the activation function layer ensures the non-linearity of a multi-layer network, and the last layer of network output generally uses an activation function to map the image features to the classification category. After the network outputs the results, it will carry out backpropagation according to the quality of the output results (generally, the loss function such as cross-entropy is used to judge). In the backpropagation process, the network parameters will implement gradient descent, improving the network parameters in the direction that can improve the model effect.

### 2.2. U-Net and FCN

**Figure 1:**
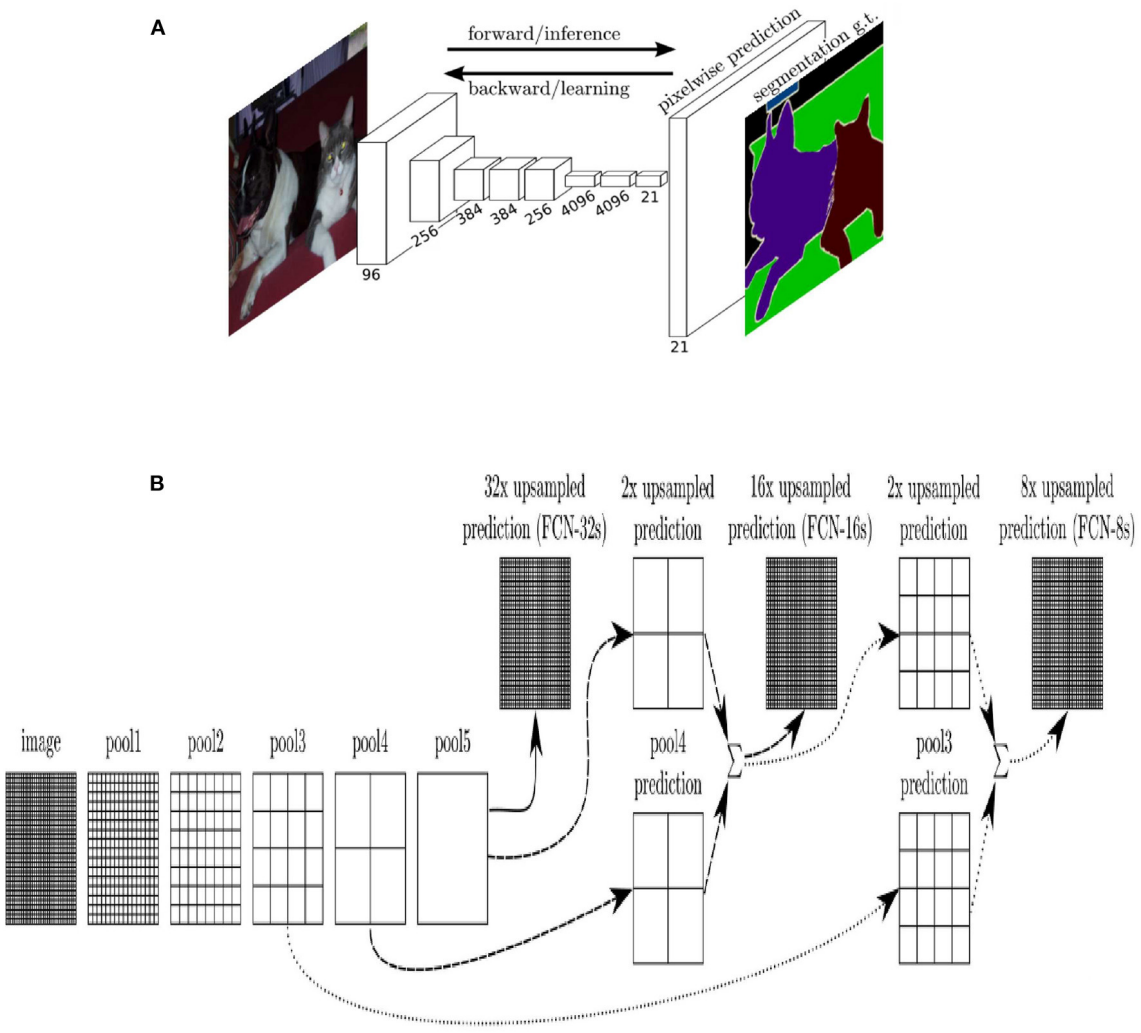
**(A)** FCN model with VGG as backbone, **(B)** skip layer of FCN: FCN-32s means output image is recovered by up-sampling directly, which has the lowest precision because of the lack of feature fusion. FCN-16s merge the feature map extracted from the last network with the feature map extracted from the previous layer before the output image is recovered. FCN-8s merge one more feature map than 16s, which has the highest precision.

There are many excellent models for semantic segmentation tasks, such as DeepLabV3 (Chen et al., 2017), hrnet (Wang et al., 2020), Transformer (Strudel et al., 2021), etc. However, the original design concept of the segmentation model comes from FCN, which first proposed to use the full convolution layer instead of the full connection layer as the activation function of output results and transform the output results of the neural network from a simple one-dimensional probability vector to a two-dimensional probability matrix, that is, segmentation accurate to the pixel level. FCN proposed restoring image size by deconvolution operation and adopted skip Layer operation. This method of concatenating high-dimensional and low-dimensional features profoundly influenced the subsequent semantic segmentation model. The FCN network structure and skip layer are shown in Figure 1.

**Figure 2:**
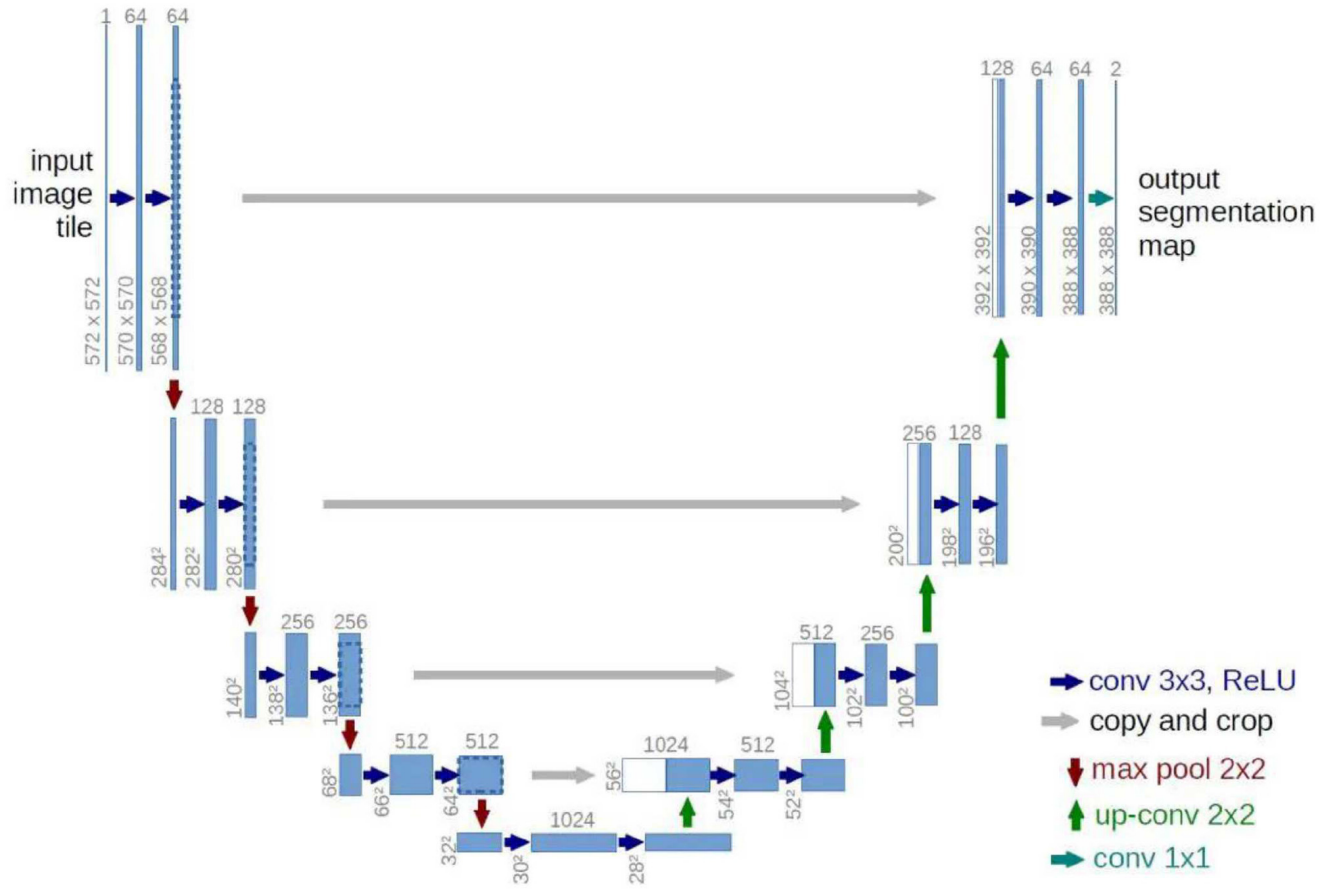
U-Net model: classical symmetric codec structure with feature concatenate.

After FCN, another model with strong influence is U-Net. U-Net model has been improved based on FCN but makes its design abandoned to use the VGG network as the backbone. Instead, a four-layer symmetrical codec structure is designed, and concatenate link is added between each layer of codec structure. Not using deep networks as backbone seems to be an effective measure to avoid overfitting in small datasets. Even at present, U-Net still has many application scenarios in a small sample environment, such as medical image segmentation. The U-net network structure is shown in Figure 2.

### 2.3. The Effect of Convolution Kernels Size

**Figure 3:**
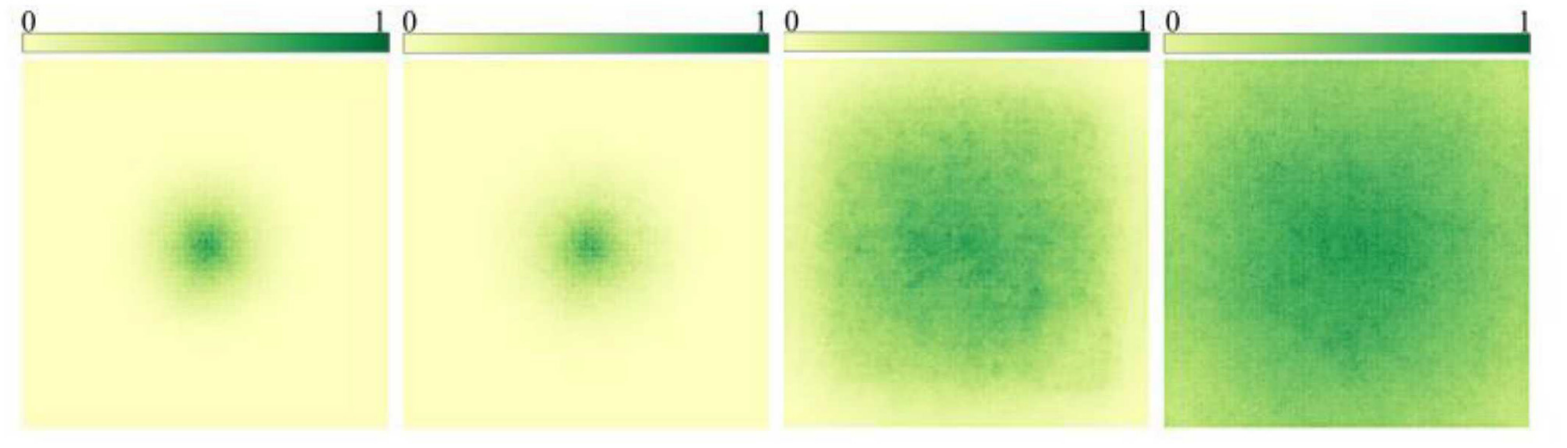
The change of receptive field caused by the change of convolution kernel size: these images are the results of a study by RepLKNet. The first two pictures are compared and superimposed with multiple small convolution checks for the improvement of receptive field, which shows that there is not much effect. The last two pictures are the effect caused by increasing the size of the convolution kernel, which can be seen as significant.

After GoogLeNet Inception V2 (Ioffe and Szegedy, 2015) proposed the idea of using multiple small-size convolutional kernels instead of large-size convolutional kernels to reduce the number of parameters while keeping the size of the receptive field unchanged, the mainstream semantic segmentation models do not use large-size convolutional check images for feature extraction. Instead, multiple 3 × 3 convolution kernels are stacked to achieve a similar effect. However, Ding et al. (2022) proposed a new idea by RepLKNet in CVPR 2022. Stacking small convolutional kernels to replace large convolutional kernels only maintains the consistency of the superficial receptive fields, but the features extracted from the two are different. The utility of large convolutional kernels in large images is significantly stronger than stacking small convolutional kernels, shown in Figure 3.

Furthermore, the problem of parameter explosion caused by a large convolution kernel can be avoided by depthwise separable convolution. Depthwise separable convolution means splitting an ordinary convolution into depthwise and pointwise convolution. This operation has been well used in the DeepLab series and can effectively reduce the number of parameters.

## 3. Method

The design ideas of our model are derived from U-NET and RepLKNet, and we adopt a coding-decoding structure similar to U-NET, as well as the large convolution kernel and Re-parameterizing mentioned in RepLKNet, but improve it for our downstream tasks. First, instead of directly inputting the whole image into the network, we split the RGB three channels of the original image and input them into the network, respectively to extract features. The network can find the color channel containing the most information in the sonar image with a relatively single overall color and focus on its weight. Then we use large convolution kernels for feature extraction, add channels with small convolution kernels in parallel for compensation, and finally output image segmentation results.

### 3.1. Noise Reduction Filter

The side-scan sonar image noise disturbance is serious compared with the camera image, so the training process needs noise filtering. Gaussian filter is often used for denoise of side-scan sonar images, whose implementation depends on the Gaussian template window, divided by the Gaussian function according to the distance between the elements in the window and the center element obtained. The expression is as follows:


(1) $$\begin{array}{r} H_{ij}=\frac{1}{2\pi \sigma^{2}}e^{-\frac{x^{2}+y^{2}}{2\sigma^{2}}} \end{array}$$

But the Gaussian filter equation belongs to the isotropic diffusion equation, it may destroy edge features. So we use Perona-Malik diffusion equation to substitute Gauss equation:


(2) $$\{\begin{array}{ll} \frac{\partial u}{\partial t}=div[g(|\nabla u|)\nabla u] & \;\\ u(x,y,0)=u_{0}(x,y), & (x,y)\in R\\ g(\nabla u)=\frac{1}{1+\frac{|\nabla u{|}^{2}}{k^{2}}} & \; \end{array}$$

K is constant. The size of the diffusion coefficient of the diffusion equation is determined from the gradient of the image: |∇*u*|, which is used to judge whether it is an image edge.

### 3.2. Depthwise Separable Convolution

**Figure 4:**
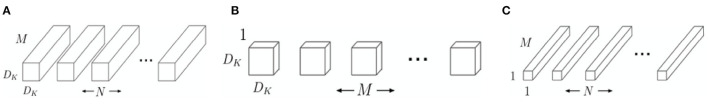
Typical conv with depthwise conv. **(A)** Typical conv, **(B)** depthwise processing, and **(C)** pointwise processing.

The concept of depthwise separable convolution was first proposed by MobileNet (Howard et al., 2017), which is shown in Figure 4. It puts forward a new idea: the standard convolution operation is decomposed into two processes: Depthwise and Pointwise. Assuming that an input image with a size of 12 × 12 × 3 is taken as the input, convolution is carried out with a convolution kernel of 5 × 5 × 3, and 128 channels of output results are expected to be obtained, the total number of parameters of this convolution operation is 9,600.

In contrast, the Depthwise process is used first. Three 5 × 5 × 1 convolutions are used to convolution the three channels of the input image, respectively, and then superposition to obtain the output result of 8 × 8 × 3. Then comes the Pointwise process, that is, 128 convolution kernels, which have the size of 1 × 1 × 3, are used to convolve the feature graph of 8 × 8 × 3 for channel expansion. Finally, the output result with the same size as the conventional convolution is obtained, but the parameter number of depth separable convolution is only 5 × 5 × 3 + 1 × 1 × 3 × 128 = 469.

Depth separable convolution reduces the number of parameters by increasing the number of network layers, which does not necessarily play a positive role in the network of small convolution kernels. However, for large convolution kernels, this operation is essential. In addition, it is noted in the article of RepLKNet that current deep learning frameworks such as PyTorch do not support DW convolution very well, so the author of RepLKNet designed a more efficient implementation based on PyTorch (Ding, 2022) [The authors claim that the MegEngine (Megengine, 2020) framework based implementation is more efficient. However, we have not used it in this article and the network architecture is still PyTorch based].

### 3.3. Multi-Scale Feature Fusion

At present, feature fusion technology of different scales is an indispensable part of the semantic segmentation model, such as skip layer of FCN, concatenate structure of U-Net, ASPP module using dilated convolution in Deeplab. Article of RepLKNet proposed a structural re-parameterization method. In the training process, a parallel feature extraction channel using a smaller convolution kernel with 3 × 3 is constructed for a large convolution kernel (e.g., 31 × 31). After training, it is added to the large convolution kernel through linear transformations. In this way, the large convolution kernel can capture small-scale features and improve network performance. In essence, it is still a multi-scale feature fusion method.

Since we do not have a large amount of training data like the RepLKNet model, we cannot use super large convolution kernels (e.g., 31 × 31) and a deep network structure similar to RepLKNet. Therefore, we do not directly add the parameters of small convolution kernels into large convolution kernels, which will destroy the performance of large convolution kernels, but adopt the concatenate method. The features extracted from large and small convolution kernels are combined in the decoder stage to achieve multi-scale feature fusion.

### 3.4. Model Structure

**Figure 5:**
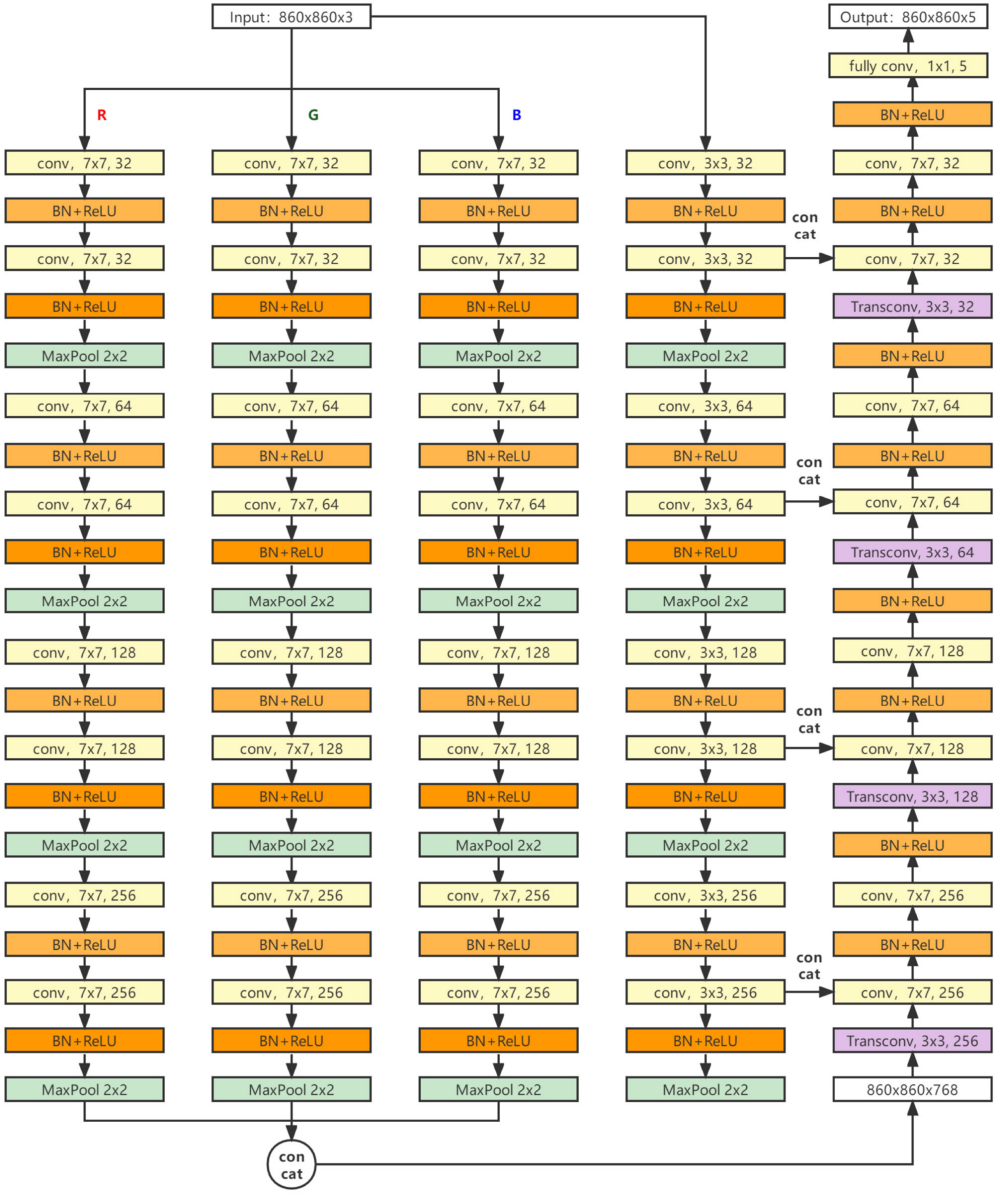
The structure of our model.

The structure of our model is shown in Figure 5. The main body is a four-layer encoder and a four-layer decoder. The data enter the encoder after noise reduction, enter the decoder after feature extraction of four layers, and then go through four layers of convolution and up-sampling. Finally, the segmentation result of the network is output after going through a full convolution layer.

The encoder part consists of four parallel feature extraction links, three of which have the same structure, and the input of each link is one of the RGB three channels of the original input image. Each link contains four layers, and each layer contains two feature extraction modules with the size of 7 × 7 convolution kernels (DW operation has been used to replace the conventional convolution mode, and the padding operation is carried out to ensure the image size remains unchanged). The number of output channels in each layer is 32, 64, 128, and 256. The output of the last three links will concatenate and output a 768 channel heat map.

The other independent link input is all channels of the original input image, and each layer contains two feature extraction modules with a 3 × 3 convolution kernel size. The conventional convolution operation is adopted, and the number of channels output by each layer is the same as that of the other three links.

The input of the decoder is the heat map of 768 channels, which is reduced to 256 channels after up-sampling by deconvolution, and then concatenated with the data of the fourth link (the link using a small convolution kernel) to obtain the feature map of 512 channels. Then, the feature map goes through two feature extraction modules with the size of 7 × 7 convolution kernels. After four cycles of such operations, the final segmentation result is output through the full convolution layer.

## 4. Experiment and Analysis

All experiments are conducted with Intel Core i9-10900F CPU@2.8 GHz x 20, 64 GB RAM, NVidia Geforce 3090 GPU, 24 GB of video memory, by CUDA Toolkit 11.3, CUDNN V8.2.1, Python 3.6, PyTorch-GPU 1.10.1, Ubuntu18.04.operating system.

### 4.1. Dataset Collection

**Figure 6:**
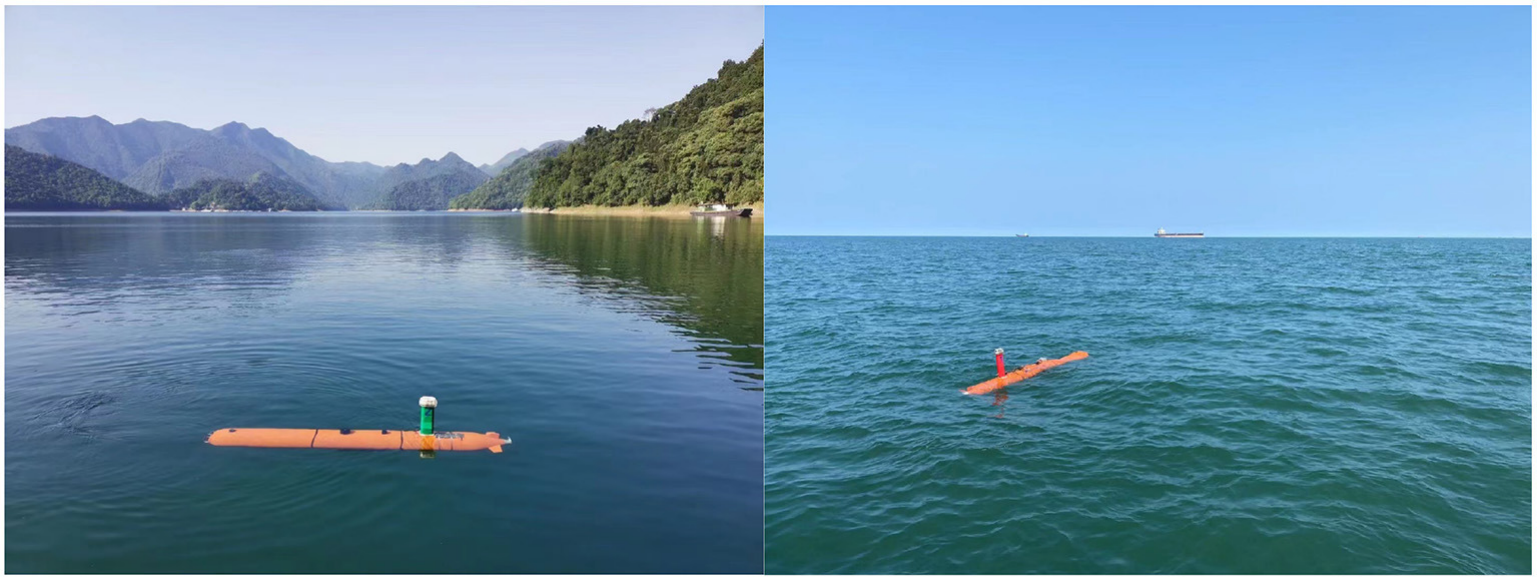
The AUV was developed by the team, the acquisition process is carried out by three AUVs simultaneously.

**Figure 7:**
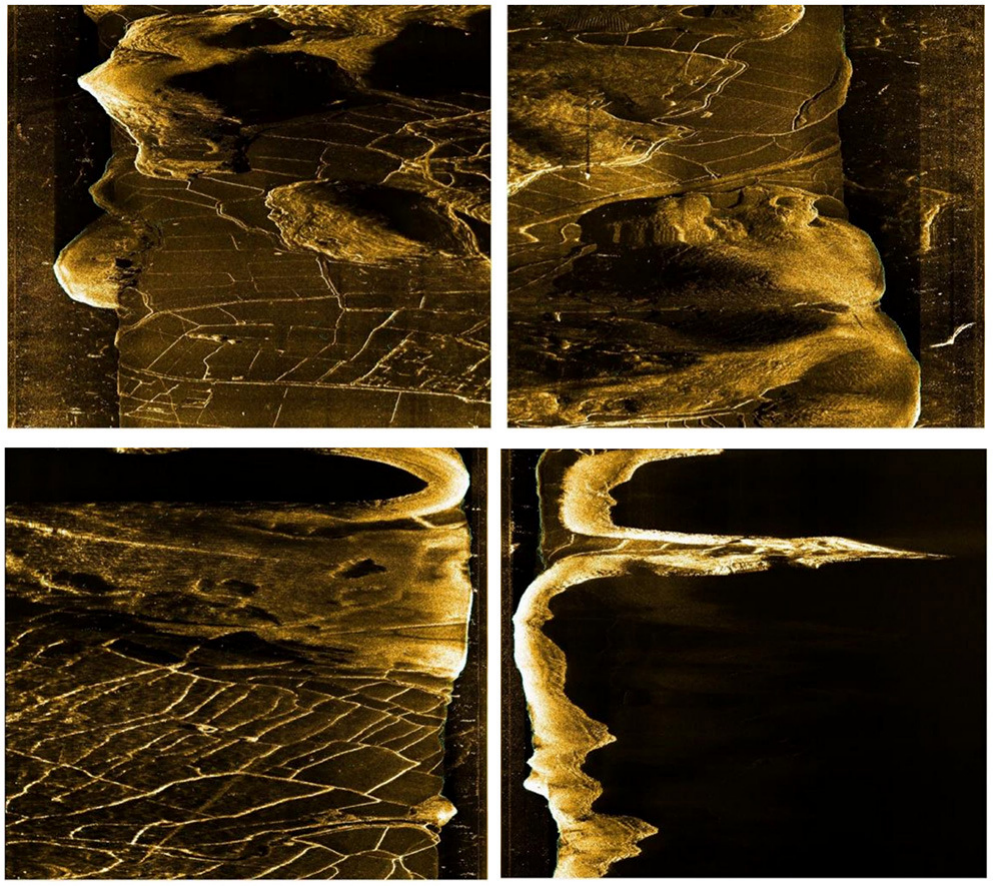
The origin sonar image (each sonar image is cropped down the middle into two images).

The sonar image dataset was collected by HYDRO 3060 side-scan sonar at Qiandao Lake, Jiande, Hangzhou, China (The unclassified portion has been uploaded to GitHub: https://github.com/YDY-andy/Sonar-dataset). The collected sonar data is processed by software from XTF form to waterfall stream video, and then the sonar data in image form is obtained through frame by frame sampling. The sonar equipment is equipped with the AUV developed and manufactured by our team. In addition, THE AUV is also equipped with inertial navigation, Doppler, GPS, and an ultra-short baseline positioning system, which can cruise by manual remote control or follow the established program trajectory. AUV collects all data in this paper, and the collection process adopts a slow and constant speed cruise. The height is 10 m underwater, and the maximum distance from the bottom is 50 m. The AUV used for data collection are shown in the Figure 6. The image data size is 960 × 900 pixels. Original sonar images are shown in Figure 7.

In moving, the side-scan sonar emits sound waves to both sides and constructs an image according to the echo intensity. The higher the echo intensity is, the higher the target's brightness, such as stones and metals, while the water part and the covered part will be presented as black parts in the image because there is no echo.

**Figure 8:**
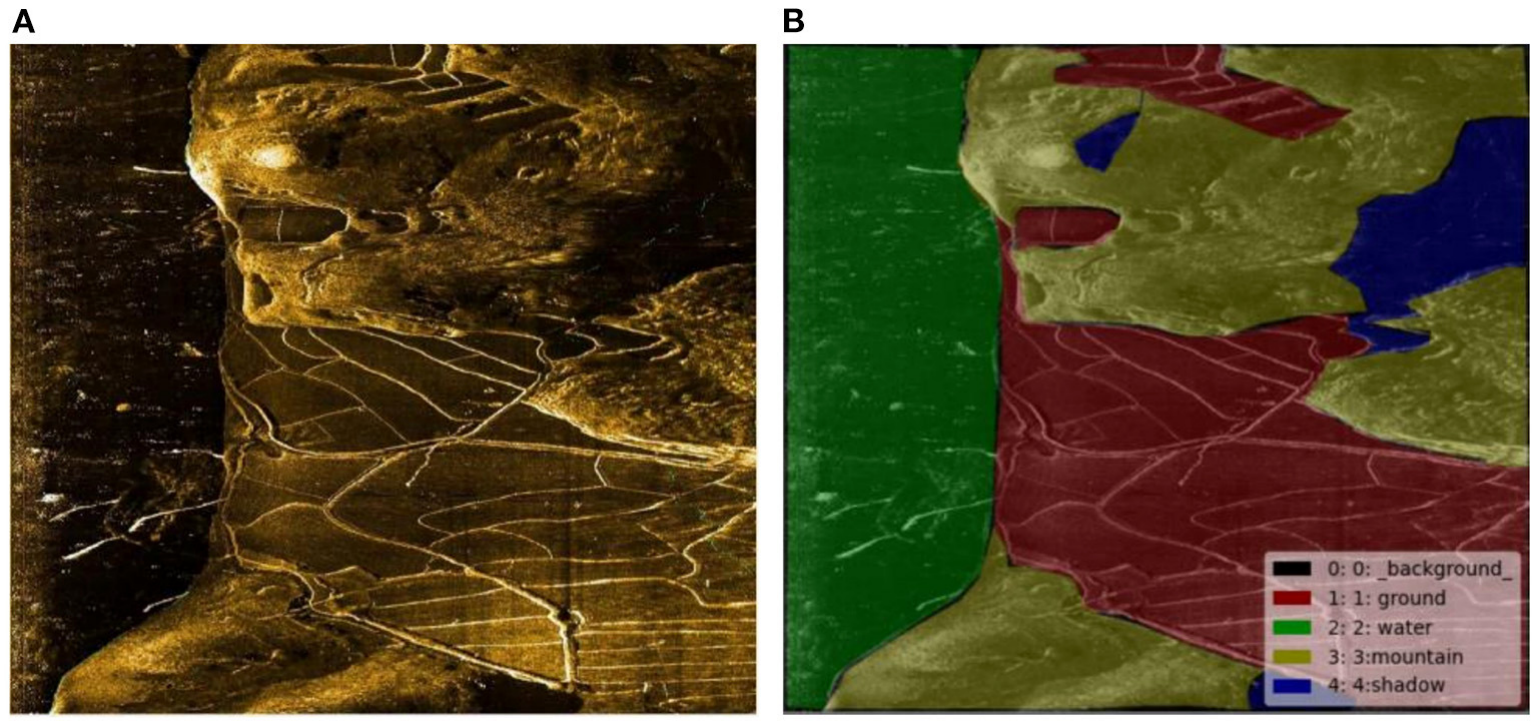
**(A)** original image and **(B)** label.

Neural network model training needs data labeled as ground truth, so sonar data must be marked. The LabelMe label software developed by MIT is used as the image label tool. A total of five categories of sonar data were labeled: (1) water; (2) Mountain part; (3) Land; (4) Shadow part; (5) Unmarked areas (background). Unmarked areas mainly refer to the remaining fragment region of the image after being marked by the first four categories. Marked images are shown in Figure 8.

### 4.2. Data Augmentation

**Figure 9:**
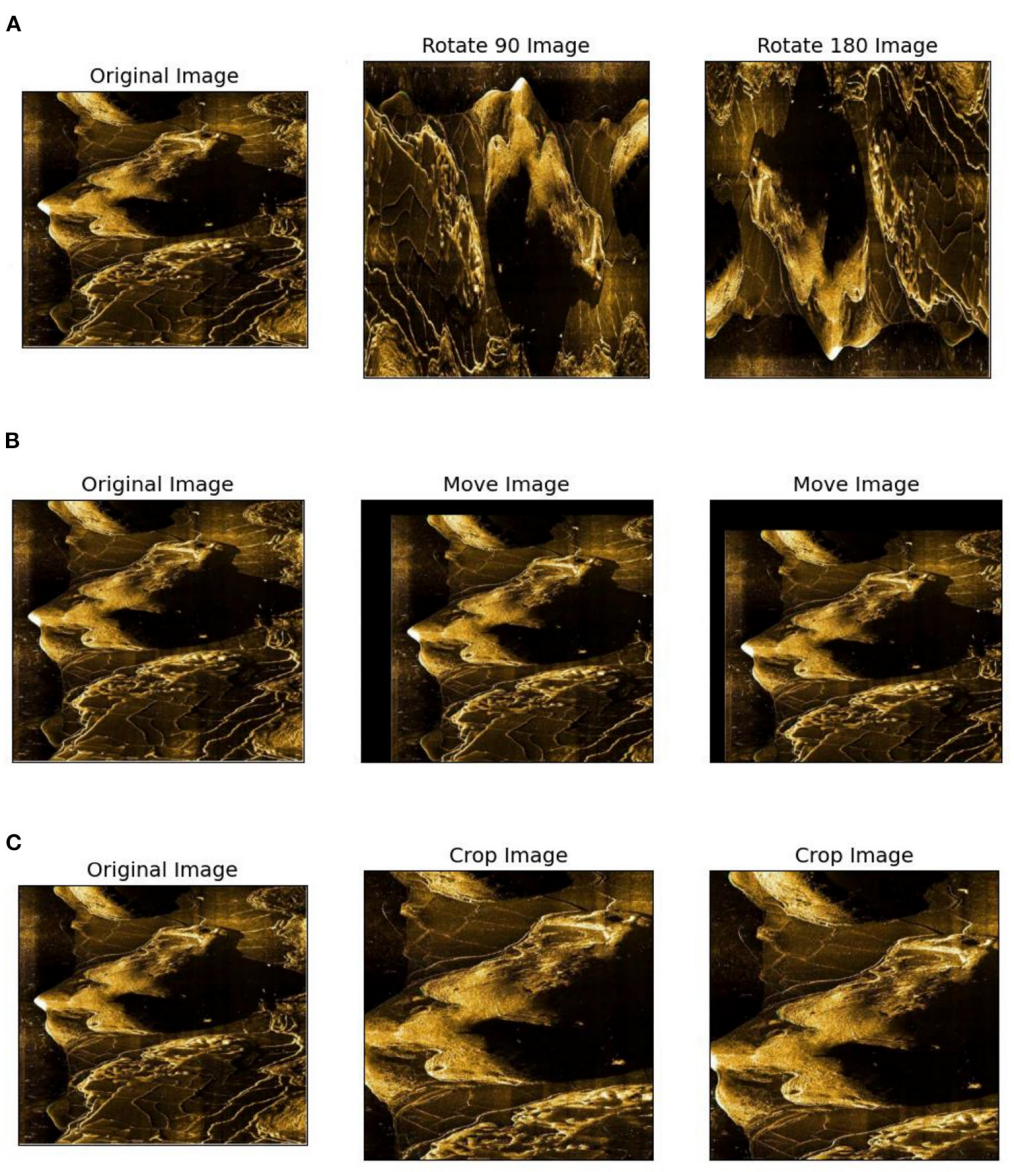
Data augmentation. **(A)** Image inversion, **(B)** Image panning, and **(C)** Random crop.

Since the sonar images collected are limited and belong to a small sample data set, the data augmentation method expands the number of samples to prevent the model from over-fitting. We use the following methods, which are shown in Figure 9, to extend the data set.

1. Image inversion is the simplest way to augment data by inverting the image horizontally and vertically to get new data.
2. Image panning. The panning distance of the image in different directions is controlled by a random number to generate new data (the translation distance should not be too large; otherwise, the data availability will be damaged).
3. Random crop. The original image was 960 × 900 pixels, cropped to 860 × 860 with a random clipping area.

Since the model itself will extract the features of the three color channels of the original image, no color augmentation is used to avoid damaging the color consistency of the original image. The original data set contained 310 sonar images with a size of 960 × 900, and 1,240 sonar images with a size of 860 × 860 were obtained after expanding the data enhancement method. Sixty percent of them were randomly selected as training datasets, 20% as validation datasets, and 20% as test datasets.

### 4.3. Verification Indicators

In order to measure the performance of the model, the number of parameters, model complexity, required memory, and model accuracy was used to judge. The model complexity indicator uses FLOPs, which refer to floating-point Operations. The calculation formula of the convolution layer FLOPs of the convolutional network is as follows:


(3) $$\begin{array}{r} FLOPs=\left(2c_{in}k^{2}-1\right)HWc_{out} \end{array}$$

*c*_*in*_ and*c*_*out*_ represents the number of input and output channels in the convolution layer, and k represents the size of the convolution kernel. The size of the output feature graph is H × W.

OA (Overall accuracy) and MIoU (Mean Intersection over Union) will measure the model accuracy. The calculation formula of OA is as follows:


(4) $$\begin{array}{r} ACC=\frac{TP+TN}{TP+TN+FP+FN} \end{array}$$

TP, TN, FP, FN means True Positive (positive sample is judged as a positive sample), True Negative (negative sample is judged as a negative sample), False Positive (negative sample is misjudged as a positive sample), False Negative (positive sample is misjudged as a negative sample).

The calculation formula of MIoU is as follows:


(5) $$\begin{array}{r} MIoU=\frac{1}{k}\overset{k}{\underset{i=1}{\sum }}\frac{p\cap g}{p\cup g} \end{array}$$

P means prediction, G means ground truth.

### 4.4. Network Model Training

**Figure 10:**
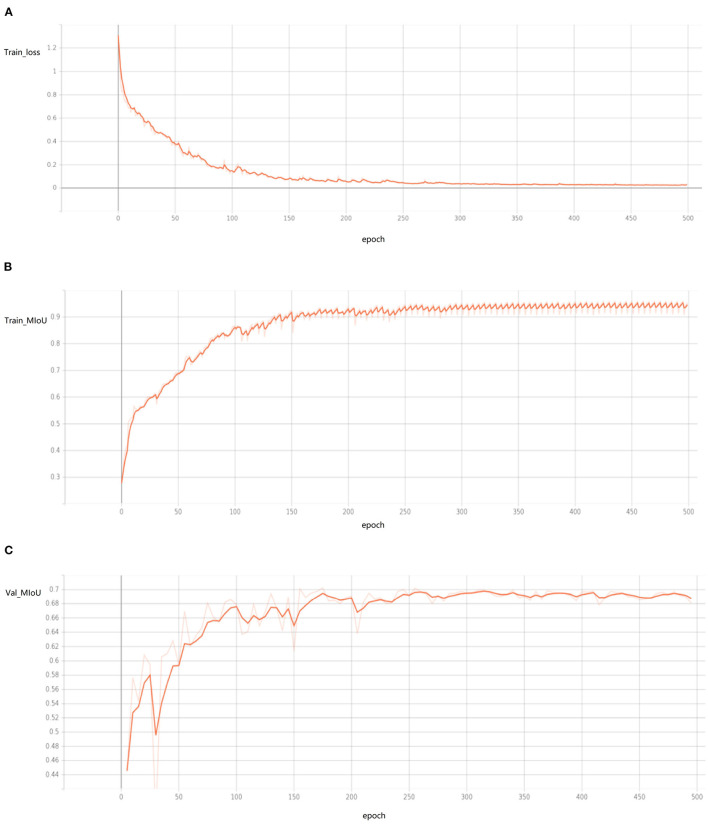
The curve of training process (drawn by Tansorboard) shows the training process of 500 epochs, but in fact the network has basically converged when there are <200 epochs. **(A)** Train loss, **(B)** train MIoU, **(C)** val MIoU.

Sonar data set is used to train network parameters. The hyperparameters used in training are shown in Table 1 (all the hyperparameters were tested and the best ones were selected). The loss function is the cross-entropy loss function. The training process is shown in Figure 10.


(6) $$\begin{array}{r} H\left(p,q\right)=-\overset{n}{\underset{i=1}{\sum }}p\left(x_{i}\right)log\left(q\left(x_{i}\right)\right) \end{array}$$

The function measures the difference between two distributions, p(x) and q(x), which means prediction and ground truth.

**Table 1:** Hyper-parameter.

**Type**	**Value**
Num of workers	8
Batch size	3
Optimizer	SGD
Learning rate	0.01
Learning policy	Poly
Step size	10,000

### 4.5. Performance and Comparison

**Figure 11:**
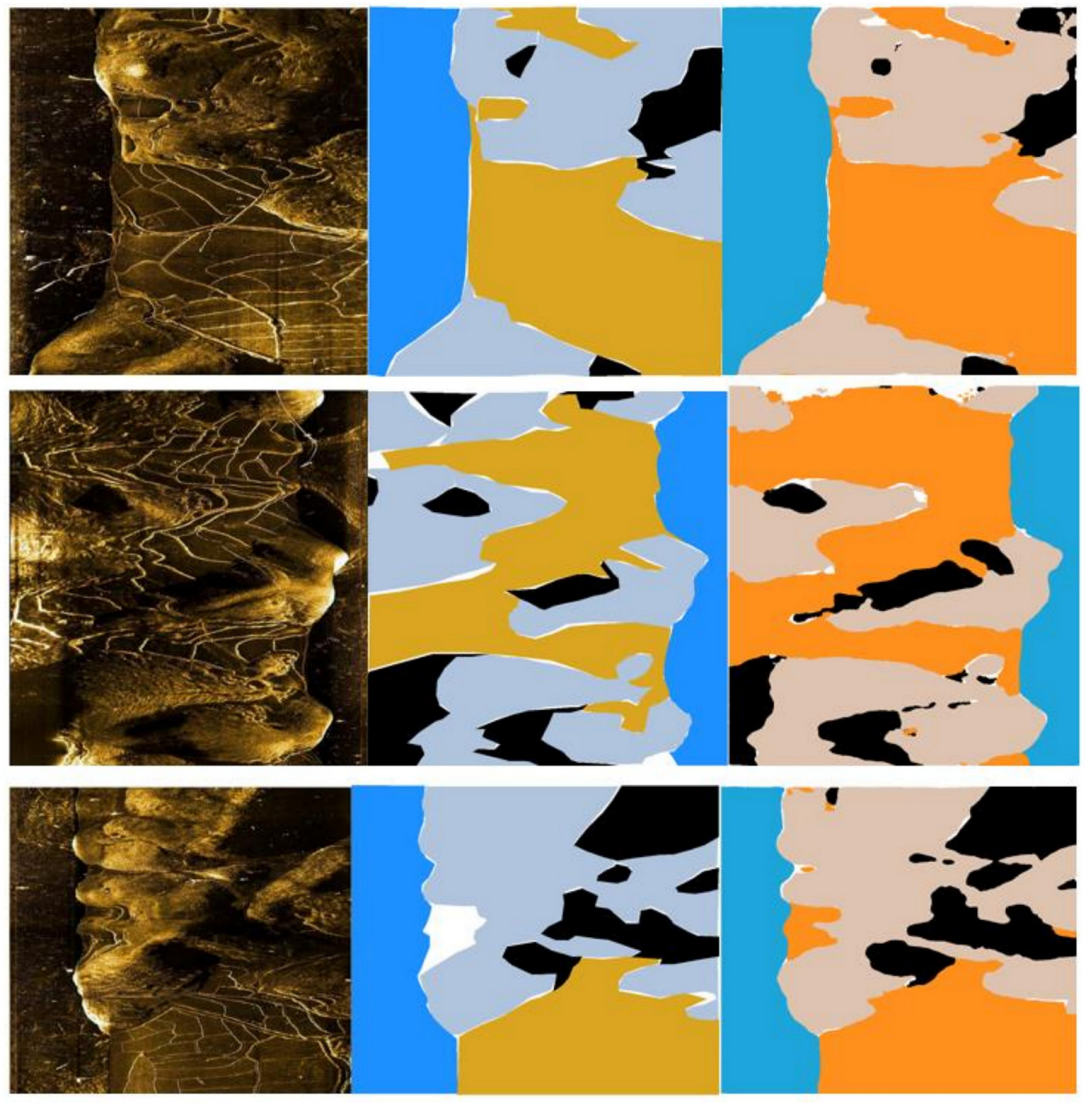
The segmentation results are shown in the figure: from left to right are the original picture, marking and training results, respectively. The colors in the figure represent the results: blue represents water, gray represents rock, yellow represents land, black represents shadow, and white represents undifferentiated background.

In the experiment, the quantitative analysis of segmentation results of U-Net, FCN, and PSPNet, which are typical lightweight networks, and our method has been conducted. The comparison results are shown in the table, and the recovered images are shown in Figure 11.

The results shown in Table 2 showed that the OA and MIOU of our model in the dataset were 0.87125 and 0.70994, which were the highest in all the four models. The total number of parameters was 13,117,135, which was also the lowest among the four models. FLOPs, at 347.52 g, were only higher than FCN, the second-to-last of the four models.

**Table 2:** Different model performance.

**Model**	**ACC**	**MIoU**	**Num of para**	**FLOPs**
FCN	0.865154	0.691115	18643845	**212.4G**
U-Net	0.862475	0.688222	34525391	487.71G
PSPNet	0.861086	0.706046	65576517	673.94G
Ours	**0.871247**	**0.709939**	**13117135**	347.52G

*Bold values mean the best values*.

We also conducted a comparison experiment of parameter tuning for our model and resized the convolution kernel from 3 × 3 to 5 × 5 to the current 7 × 7 and then to 9 × 9 and 11 × 11. Its MIoU is shown in Table 3. It can be found that the model parameters currently in use have the best performance, and the convolution kernel with the size of 7 × 7 currently in use has the best performance without increasing the amount of data and network depth. In the RepLKNet paper, however, the authors show that scaling up to 31 × 31 can improve network performance, perhaps by relying on short-cut structures, which significantly increases network depth and increases the risk of overfitting. In addition, even if DW convolution is adopted, the rapid increase of parameters will bring difficulties to devise transplantation. Therefore, we believe that the scale of the 7 × 7 convolution kernel is an appropriate parameter setting.

**Table 3:** Model performance with different kernel size.

**Size**	**ACC**	**MIoU**
3 × 3	0.852023	0.679179
5 × 5	0.860964	0.694266
7 × 7	**0.871247**	**0.709939**
9 × 9	0861946	0.692680
11 × 11	0.862763	0.687616

*Bold values mean the best values*.

The decoder uses a 3 × 3 deconvolution operation to restore the image. The experiment proves that the size of the deconvolution convolution kernel does not influence the final result, so the most straightforward 3 × 3 scale is adopted.

We also tried feature fusion which is not applicable to parallel small convolution kernel lines, shown in Table 4, and MIoU plummeted to about 0.61. In addition, MIoU is reduced to about 0.67 when the whole image is extracted as a whole instead of the RGB three-channel feature extraction method. Therefore, it can be concluded that feature fusion operations of convolution kernels of different sizes and the RGB three-channel feature extraction method are essential.

**Table 4:** Model performance with different structure.

**Model**	**ACC**	**MIoU**
Prototype	**0.871247**	**0.709939**
No parallel small channels	0.834063	0.614298
No multi channels	0.845060	0.670283

*Bold values mean the best values*.

A series of experimental results show that multi-scale feature acquisition channels are significant in networks with limited depth (in small data sets, large depth networks are prone to overfitting), which is why many network models now always have a dimension of expansion in depth and width. At the same time, the combination of channel dimension and scale dimension can better enhance the segmentation accuracy of the network. For the sequence information, the feature of time dimension can also be added to form the feature fusion of space, time, and color. Sonar images belong to the waterfall video capture results, which have the potential to add time dimension features. We will consider introducing a sequential neural network for feature extraction in subsequent studies.

## 5. Conclusion

AUV navigation has not only relied on inertial navigation but also improved its accuracy through the joint action of various sensors. However, most current studies have not fully utilized the semantic information of images collected by side-scan sonar. This study proposes a deep learning model for image segmentation of side-scan sonar. The model adopts the codec structure to extract the features of the RGB three channels of the image, and finally, the fusion is carried out. The weight assignment of specific channels in sonar images with single-color information is considered emphatically. At the same time, large convolution kernels were used to increase the receptive field, and small convolution kernels were added for feature fusion to ensure the richness of the feature scale. We show that the model has low computational cost and flexibility in the sonar image segmentation problem. The experimental results show that our model has certain advantages in many indicators. It can provide semantic segmentation results of side-scan sonar images in AUV navigation to assist location matching. In the future, we will consider adding channel fusion between multiple channels and adding shortcut modules to increase the convolution kernel's size further.

## Data Availability Statement

The raw data supporting the conclusions of this article will be made available by the authors, without undue reservation.

## Author Contributions

DY and FZ conceived the study and put forward the methodology. CC and CW performed the data collection and pre-processing. DY carried out the software for the experiments and wrote the first draft of the manuscript. FZ and GP reviewed and edited the manuscript. All authors read and agreed to the published version of the manuscript.

## Funding

This study was supported by the National Natural Science Foundation of China (52171322), the National Key Research and Development Program (2020YFB1313200), and the Fundamental Research Funds for the Central Universities (D5000210944).

## Conflict of Interest

The authors declare that the research was conducted in the absence of any commercial or financial relationships that could be construed as a potential conflict of interest.

## Publisher's Note

All claims expressed in this article are solely those of the authors and do not necessarily represent those of their affiliated organizations, or those of the publisher, the editors and the reviewers. Any product that may be evaluated in this article, or claim that may be made by its manufacturer, is not guaranteed or endorsed by the publisher.
